# Supplementary material for: Non-invasive assessment of liver disease in rats using multiparametric magnetic resonance imaging: a feasibility study
Source: Biol Open. 2018 Jun 18;7(7):bio033910. doi: 10.1242/bio.033910 (PMC6078340; doi:10.1242/bio.033910)
Supplement: Supplementary information [file biolopen-7-033910-s1.pdf]

© 2018. Published by The Company of Biologists Ltd | Biology Open (2018): doi:10.1242/bio.033910: Supplementary information

FIRST PERSON

First person – Anna Hoy

First Person is a series of interviews with the first authors of a selection of papers published in Biology Open, helping early-career researchers promote themselves alongside their papers. Anna Hoy is first author on 'Non-invasive assessment of liver disease in rats using multiparametric magnetic resonance imaging: a feasibility study', published in BiO. Anna is a postdoctoral scientist in the lab of Jonathan Fallowfield at the University of Edinburgh, UK. Her research interests lie in liver disease with particular emphasis on drug and biomarker discovery and development.

What is your scientific background and the general focus of your lab?

My scientific background is in translational biology. I undertook my undergraduate studies in Poland where I studied biotechnology and molecular biology. I relocated to Scotland in 2008 and completed an MSc in instrumental analytical sciences at the Robert Gordon University, Aberdeen, for which I was awarded a distinction. During my PhD at the University of Edinburgh, I investigated the diagnostic and therapeutic role of microRNAs in *Schistosoma mansoni* infection and liver fibrosis, under the mentorship of Dr Amy Buck and Prof. Andrew MacDonald. Since 2014 I have been working as a postdoctoral researcher in the lab of Prof. Jonathan Fallowfield, on three industry–academia collaborative projects. The Fallowfield lab's research interests span the field of hepatology with studies in basic science, translational medicine and clinical studies. Key topics of research include mechanisms of liver fibrogenesis and fibrosis regression, novel diagnostic markers (especially imaging) and discovery/development of novel therapies for liver fibrosis, non-alcoholic steatohepatitis (NASH) and portal hypertension.

How would you explain the main findings of your paper to non-scientific family and friends?

Non-alcoholic fatty liver disease (NAFLD) is the most common chronic liver disease in both rich and poor countries, impacting public health and healthcare globally. There is a huge effort from the pharmaceutical industry and academia to develop and trial new therapies for NAFLD and liver fibrosis in general; however, a lack of accurate and reliable non-invasive methods for assessing liver disease is a major obstacle to the drug development process. Microscopic examination of liver biopsy remains the gold standard despite its limitations such as sampling error (where only a small fragment of liver is taken), high cost as well as risk of complications. MRI is a non-invasive technique that can be used clinically to examine the composition and structure of tissues. A recently developed clinical MRI tool, Liver*MultiScan*™, assesses hepatic fat, fibro-inflammatory injury and iron and is being integrated into clinical trials for liver disease. The aim of our study was to develop and apply an analogue MRI protocol using a small animal scanner that can be used in preclinical drug testing. We demonstrated that multiparametric liver MRI is feasible within a realistic time frame and showed good diagnostic accuracy in animal models of liver disease. MRI parameters correlated with gold standard microscopic assessment of liver tissue samples, especially characteristics of fatty liver disease. Further validation in longitudinal animal studies is now required.

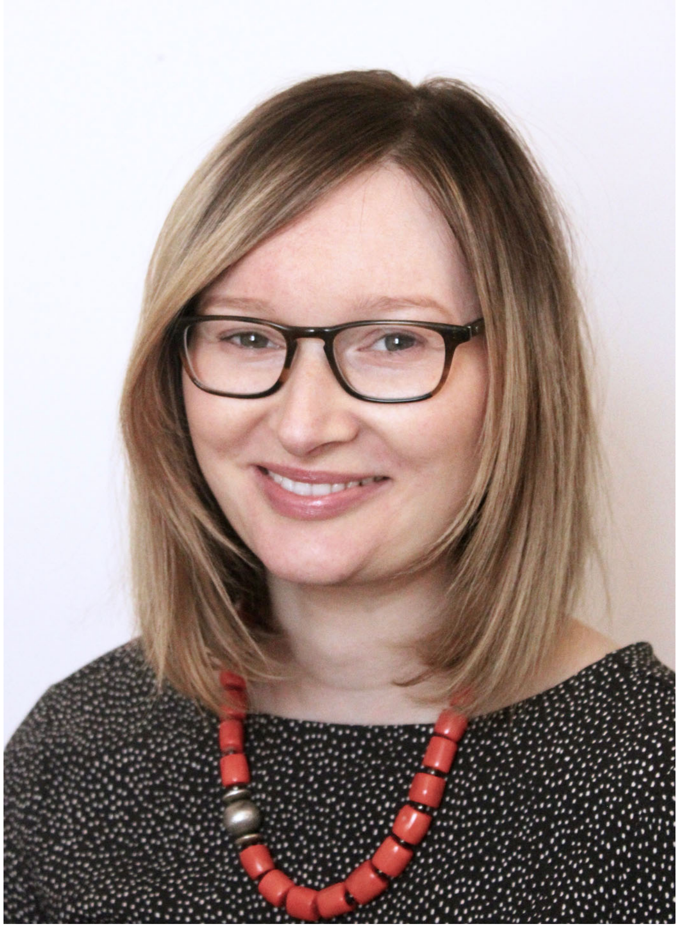

Anna Hoy

"Our findings indicate that multiparametric liver MRI could potentially refine the way we currently approach testing of therapeutic candidates in preclinical models."

What are the potential implications of these results for your field of research?

Our findings indicate that multiparametric liver MRI could potentially refine the way we currently approach testing of therapeutic candidates in preclinical models. Firstly, multiparametric MRI could be used as a quantitative and objective method to stratify liver disease

Anna Hoy's contact details: MRC/University of Edinburgh Centre for Inflammation Research, Queen's Medical Research Institute, 47 Little France Crescent, Edinburgh EH16 4TJ, UK.

E-mail: anna.m.j.hoy@gmail.com

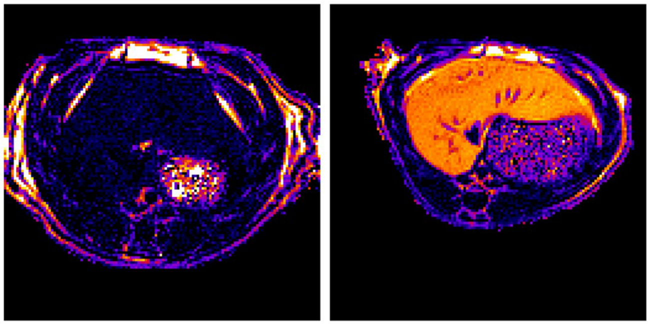

MRI map showing fat distribution in a healthy rat (left) and rat on a research diet that results in fatty liver disease (right).

severity before exposing animals to a drug treatment. Secondly, multiparametric MRI could potentially be used longitudinally to evaluate the effect of an antifibrotic or anti-NASH intervention in individual experimental animals, allowing each to serve as its own control, and thereby allowing determination of the distribution of effects in an experimental population. This approach would also address the 3Rs (refinement, reduction, replacement) of humane animal experimentation.

What changes do you think could improve the professional lives of early-career scientists?

The biggest issues early-career scientists face are limited research funding and job insecurity, and their impact on personal life. Despite our considerable efforts and long working hours, short-term contracts (especially those of one year duration) simply do not give enough time to generate high-impact data, making it difficult to publish and obtain further funding. This puts huge pressure on scientists and sadly pushes talented people to leave academia in pursuit of permanent positions elsewhere. Increased availability of university funding allowing permanent teaching positions and longer research contracts would significantly improve the professional lives of early-career scientists.

What's next for you?

I am currently on maternity leave looking after my newborn twins and their big sister. Since my current contract ends shortly I am exploring both academic as well as non-academic career pathways. I am excited about the many possibilities for applying my experience, knowledge and skills.

Reference

Hoy, A. M., McDonald, N., Lennen, R. J., Milanesi, M., Herlihy, A. H., Kendall, T. J., Mungall, W., Gyngell, M., Banerjee, R., Janiczek, R. L., Murphy, P. S., Jansen, M. A. and Fallowfield, J. A. (2018). Non-invasive assessment of liver disease in rats using multiparametric magnetic resonance imaging: a feasibility study. *Biol. Open* **7**: bio033910.
